# Whither Are We Drifting?

**Published:** 1905-05

**Authors:** 


### Editorial.

### WHITHER ARE WE DRIFTING?

The business man at certain periods takes account of his affairs and bases his future prospects on his present standing and upon the general tendencies of the world at large and carefully measures the possibilities. The professional man has a similar duty and must follow these well-understood business principles, and adapt them not wholly to financial success, but to that broader life that should be part of his professional heritage and which is part of his duty to cherish.

Dentistry, it seems to the writer, is passing through a phase of experiences that may end in its advantage, or it may result in serious loss. Are we standing upon the brink of a commercial maëlstrom, dashing and whirling, inviting to eventual destruction? or are the unpleasant signs manifest but the distant wind clouds stirring the waters and presaging the storm, destined to clear the atmosphere and restore to more healthful surroundings?

The indications in the dental world point to a condition far from satisfactory. It seems as though we are drifting more and more away from the spirit which actuates and which is the soul of all professions. The commercial taint, the sordid influence of wealth, hoped for or attained, has entered our ranks to an extent that promises to undermine the very foundations of our professional life. The evidences of this are everywhere. The poison is so insidious that few realize the hold it has taken upon the many that in part compose the twenty-five thousand engaged in the practice of dentistry. This large body would probably treat with scorn the charge, made individually, that they were not professional, or that they were dragging down dentistry again into the mire from which it has been painfully and laboriously exhumed. Yet it must be recognized that the spirit of gain is rapidly demoralizing the dental practitioners of this country.

The signs of this are manifest in various directions, but perhaps more notably in the character of certain articles prepared to be read before societies. These are more devoted to the consideration of

*fees*, and the writers, and those who take part in the discussions, grow argumentative in regard to the importance of establishing a trust or its equivalent. They are not satisfied with the fees obtained in their several localities. Comparisons are made between those received for dental operations and those recognized as legitimate in medicine and law. It must be acknowledged that remuneration for services rendered is not up to the professional standard, nor in accordance with the standard dentistry should occupy as one of the most important branches of the healing art. The matter has, however, a deeper bearing than is usually recognized in papers, and the remedy is not to be found in binding resolutions or trusts built upon these.

The men who thus eloquently portray their financial losses apparently lose sight of the fact that the tendency of which they complain is the result of non-professional practices, of which the papers alluded to are, in part, a painful exhibit. The man who respects himself and his profession will arrange the charges for service with a due regard to his own higher interests and to the honor of the profession of which he is a member. Who ever hears of a medical practitioner rushing into print and complaining that he finds it impossible to convince his patients that he has not overcharged them, and asking his *confrères* to establish a fee bill that all practising medicine must maintain? The thought of money in connection with professional service is, to that extent, a lowering of the standard of the calling. The time was when the honorarium was left to the patient and to the feelings of gratitude and extent of the purse. It was held to be undignified to present a bill, and patients resented such a departure from professional methods. This feeling still prevails in many communities. The influence of money is too powerful in our modern life to admit a higher standard than will develop into professional procedures, but it nevertheless remains true that those who think constantly of the fees at the end of the work are, to that degree, upon a lower level, and will, increasingly, drop still farther in that self-respect so necessary to loftier aspirations.

Individuals may not be so much to blame for this condition of mind, for they have notable examples furnished them by all the dental organizations of this country. When they see the majority of these dental societies, from the International Dental Congress to the National, State, and local associations, offering their proceedings to the highest bidder, they naturally feel that if the ethics of the representative organizations permit this, the individuals composing the rank and file should work in the same way and for the same end. A few societies are endeavoring to change, by precept and example, this ignoble practice, and if there is to come a better state of things, it must be through their unselfish work. The prospect is, however, not encouraging. The competition between the so-called trade journals for the proceedings of the several dental bodies in this country has become intense; indeed, it promises to reach the proportions of a professional scandal. Every effort is being made to influence societies. Their cupidity is appealed to in the saving of large annual fees to the individual members, as the expense of publishing the transactions will fall upon the successful trade periodical that, in this way, becomes the organ of the society, and this fact, it may be, is paraded on the cover of the journal. The societies so advertised may not regard this of any consequence, but some others look upon it as a badge of dishonor, not to the journal, but one which no professional body imbued with the highest ethical standard should permit.

It is a matter of surprise that the dental profession has been willing to thus weaken its position, and, to that extent, has made it impossible to call it a body worthy the professional name. When the dental organizations cease from this humiliating traffic, an example will be set that will do more to establish a higher grade of thought and practice than any long dissertations on ethics could hope to accomplish.

The National Dental Association has had the question before it for several years, of establishing a journal for the publication of its own proceedings. At the next annual meeting this matter will again come before that body, and it is hoped that it will find a way to meet this demand. A journal once established will force by example the subordinate societies to accept this as the organ for the publication of their proceedings, and in this way, and in no other, will the best thought of the dental profession find a proper and dignified presentation.

The true professional man has no quarrel with the trade that furnishes him with his needed supplies. These are a necessary adjunct to all the professions. The clergyman must have his publications for reference, the lawyer his books, the medical man his medical supplies and his instruments, and the dentist could not practise his work without them. The writer is not in full agreement with the able and incisive article by Dr. J. E. Weirick, of St. Paul, and transferred to the pages of the April number of this journal. He condemns societies for permitting trade exhibits at their meetings. While this has frequently been a detriment to the work of the organization, by offering a counter-attraction, it must be conceded to be an important part of dental education. The occasional abuse of a good thing is not a sufficient cause for total condemnation. Medical associations have the same thing, but they seem able to manage this better than dental societies, and find less interference with the work of their meetings.

It is in the details of professional life that we must exercise the greatest care. It is in those things that many regard as minor matters that this moral disease finds its best culture field. The practitioner who makes out his bill with every item spread out before his patient has no reason to expect that patient to have a very exalted idea of his professional standing. By this act he allies himself with the tradesman and mechanic. He should charge "for professional services," and not descend to undignified particulars.

We are, as a profession, apparently drifting into the stream that runs to the ocean of selfish accumulations. The hope of the future lies in an educated class of young men trained to a better standard of thought and practice. These must carry dentistry out and beyond its present unsatisfactory conditions. If this hope finds no fulfilment, then, indeed, will the end justify the statement of some,—that dentistry is simply a mechanical calling, without culture and without a dignified self-respect.

